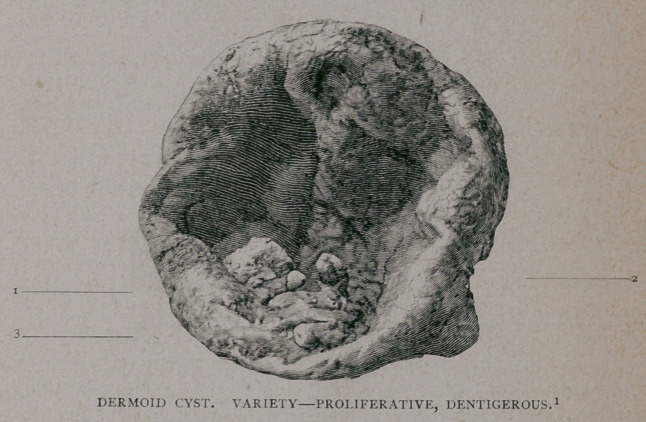# A Dermoid Cyst of the Left Ovary

**Published:** 1889-12

**Authors:** William Warren Potter, William C. Krauss

**Affiliations:** Buffalo, N. Y.; Niagara University, Buffalo, N.Y.


### A DERMOID CYST OF THE LEFT OVARY. OPERATION.—RECOVERY.

By WILLIAM WARREN POTTER, M. D., Buffalo, N. Y.

Pathological Demonstration. By William C. Krauss, M.D., of Niagara University, Buffalo, N.Y.

Dermoid Cysts, though not infrequent, are not sufficiently common, nor is their pathogenesis so well established as to render reports of isolated cases uninteresting or without value. The clinical antesection history of the following case is kindly furnished by the attending physician, Dr. J. D. Macpherson, of Akron, N. Y.:

Mrs. R——, aged 53, mother of two children, has had several miscarriages, and menstruates regularly. I saw this patient first, Oct. 16, 1888, when I found her suffering from severe pain in the cecal region, associated with marked tenderness at that point, together with tympanites and localized swelling. I made a diagnosis of obstruction of bowels, Dr. Lapp, of Clarence, in attendance at the time as consulting physician, coinciding in this opinion. The patient was kept under opium for twelve days, when her bowels moved naturally and she made a rapid and apparently full recovery. She summoned me again, April 1, 1889, when I found her suffering from severe pain in the lower part of her abdomen, temperature 104°. Under treatment for pelvic peritonitis these symptoms subsided and I discharged her from care the second time. April 14th, a week later, I was again summoned with the information that her abdomen was swelling. Upon examination I discovered a tumor rising from the pelvis which I surmised at once to be a distended bladder, as the patient informed me she had passed but a small amount of urine for some time. A catheter was introduced, but no urine was obtained. I saw the case the following day with Dr. Lapp, and upon the supposition that the tumor contained pus, a hypodermic needle was introduced, and enough fluid drawn to satisfy ourselves of the nature of its contents. We aspirated a week later, (as patient was menstruating profusely at that time), and drew off two quarts of healthy pus. The sac filled up again in about two months, and aspiration was again resorted to, with a similar result, though this time aspiration was followed by a chill and subsequent peritonitis. This time the sac filled even more rapidly than before. From the position of tumor, its inclining more to the left side, we now concluded it to be a cyst of the broad ligament, and recommended an operation to the patient and friends. Consent having been obtained the date of operation was set for August 24, 1889, and Dr. W. W. Potter, of Buffalo, N. Y., was called to make it.

With only this meager history to guide me, and as the patient was in a markedly septic condition, I concluded that I had a suppurative cyst of the broad ligament to deal with, though a positive diagnosis was not ventured. The only thing to do was to open the abdomen, with a view to a removal of whatever might be found.

Abdominal section, August 24, 1889, at 8.30 A. M. Potter, operator. Present, Drs. J. D. Macpherson, of Akron, and Dr. Henry Lapp, of Clarence, the attending and consulting physicians; also Dr. B. H. Daggett and Dr. Edward Clark, of Buffalo; Dr. A. N. Moore, of Rapids Bridge, and Dr. Martin, of Clarence.

The short median incision quickly disclosed the cyst, that was found strongly adherent in all directions. It was tapped with an ovarian trocar, and something over four quarts of pus, flocculent towards the last, were drawn. Some time was consumed in shelling out the cyst, from its stoutly adherent attachments to all the adjacent viscera, and the operation was necessarily a very dirty one. The patient seemed well-nigh collapsed by the time the cyst was released, but a free irrigation with quite hot water rallied her promptly. The abdomen was closed, with a drainage-tube left in the lower angle of the wound; and the usual dressings applied. In the evening, after the operation, her temperature rose to 100½° F., with the pulse at 130. Next morning, the temperature fell to 99° F., with the pulse below 90. The temperature lurked in the vicinity of normal, being slightly subnormal at times,

DERMOID CYST. VARIETY—PROLIFERATIVE, DENTIGEROUS.[1]

during the following week. The drainage-tube was removed on the fourth day, and the patient made a rapid and uninterrupted recovery.

To the excellent after treatment by the attending and consulting physicians, belongs much of the credit of the cure.

Upon enlarging the opening in the cyst five teeth were found, and the remains of what seemed to be a maxillary bone. The cut which appears above shows three of the teeth very clearly, the others being now in possession of one of the physicians in more immediate attendance. I leave to Dr. Krauss the histological description of the tumor.

WILLIAM WARREN POTTER.

1. The walls have been opened sufficiently, allowing the edges to be everted so as to show the interior of the cyst. The presence of teeth, three in number, can be easily recognized. Photo by W. C. K.

HISTOLOGICAL AND PATHOLOGICAL DEMONSTRATION, BY DR. KRAUSS.

Dermoid cysts have been classified under the teratomas by Virchow, in view of the number of different tissues which enter into their structure. They also come under the head of congenital or developmental cysts, taking their origin, in all probability, at some early intrauterine period.

The exact nature of the genesis of these cysts is still shrouded in mystery. The most plausible theory yet offered, and the one prone to be accepted, is that they result from a misplacement, or involution of the epil and mesoblast of the embryo, at an early period of development. The misplaced epithelial elements, contained in the stroma of the ovary or other organ, in developing produce a neoplasm, whose inner walls assume the character of the epidermis, with its structure, appendages, and, in part, its functions.

The theory, once held in good repute, that they result from some ectopic gestation, has long been discarded; the character of their contents and mode of development preclude all possibility of their being the remains of an undeveloped fetus.

The favorite seat of these cysts appears to be in the ovaries, as two-thirds of all known cases have been found in these organs. Next to the ovaries, they are most frequently encountered in the testicles, and have even been found in the mediastinum, lungs, and cranial cavity.

*Structure.*—The walls of dermoid cysts are composed of an external or muscular, and an internal or epidermal layer. All interest, however, is centered in the latter, which consists of a corium and an epidermis of flattened epithelial cells, simulating closely the structure of the skin. This layer may vary considerably in structure and appearance. Sometimes it is of a smooth epidermoidal character, or it may be nodular, with hypertrophy of the corium and epithelial cells, resembling warty excrescences. Cartilaginous tissue, irregular-shaped bony structures, as bony plates, misformed maxillary bones with alveoli set with teeth and structures corresponding to the nails, have all been met with complicating this inner layer. It may also, at times, possess the appendages and structures of the normal skin, developed to a high degree of perfection. Sebaceous glands, with secretory power, sweat glands and hair follicles, capable of performing their functions, have been found in the walls of these cysts. Nerve elements, brain tissue and unstriated muscular fibers have occasionally been observed. Cysts, with this variety of contents, have been called proliferative.

Lebert found, in half the cases of dermoid cysts, the presence of teeth, while Pauly found them in only one-sixth of the cases. They may be set in alveolar cavities, or may be attached to the walls by connective tissue bands. The number of teeth found in these cysts varies from 1-300. (Cases reported by Plocquet and Autenrieth 1807, and by Schnabel 1844). The variety may include incisors, canines, bicuspids, molars, or wisdom, perfectly developed with fang, neck, crown, enamel, tartar and cement. In the majority of cases, however, they are imperfectly developed, being irregular in shape, and incapable of being classified. Those cases of dermoid cysts in which teeth complicate the structure are designated as dentigerous.

A close histological examination of the heterogeneous elements found in the walls of dermoid cysts, discovers nothing to distinguish them from the same tissues which occur normally in the human structure.

The size of these cysts may range from that of a walnut to a man's head, depending, of course, upon their contents, which is the result of continuous activity of the glandular structures. Large masses of fatty matter, epidermal scales, cholesterine crystals, leucocytes, bunches of hair, etc., have been found enclosed within their walls.

This short sketch of the development and structure of dermoid cysts may aid somewhat in a better comprehension of the description which is to follow. It may also serve to remind the reader of the great variety of anatomical elements present in such a neoplasm, and to show how far the development of this cyst has advanced.

DESCRIPTION OF DR. POTTER'S SPECIMEN.

The cyst is slightly globular in form, with a transverse diameter of four and a half inches, and a longitudinal diameter of five and a half inches. (In the fresh state, these figures were undoubtedly much increased, the action of alcohol tending to contract the tissues.) The external surface presents a ragged, uneven appearance, with long irregular shreds of connective tissue, having once served as attachments to the neighboring organs. The cyst does not appear to have had a pedicle, as no remnant of one is recognizable. A small fragment of the Fallopian tube, much distended, is found attached to the external surface.

The external or muscular coat is variable in thickness. In some places nothing but a broad aponeurotic structure is present, which soon expands into dense, muscular layers, from one-sixteenth to one-quarter inch in thickness. These muscular fibers have a more or less circular arrangement, and are of the plain non-striated variety. Masses of adipose tissue, blood-vessels of different sizes, and several nodules of cartilage, the size and form of a small bean, were found imbedded in this muscular coat.

The internal, or epidermal, layer claims most of our attention. It is smooth, soft, velvety at places; then it takes on a rugged nodular appearance. In some places, small cauliflower-like excrescences, of considerable area, are present. The thickness of this layer is also variable, depending upon the condition of the corium and epithelial cells, whether in state of hypertrophy or not. Scrapings from the surface of this layer, examined microscopically, show flattened epithelial cells, fat globules, leucocytes, and detritus.

Near the original opening of the cyst are grouped a number of well-formed teeth, some set in alveolar cavities, others joined to the wall of the cyst by connective tissue fibers. The number of teeth originally present was five; subsequent to the operation, two became detached, and were removed. In the walls of the cyst, a flattened triangular bony plate, with sharp edges, is found, projecting through the soft structure. Leading down to this bony plate are two alveolar cavities, whose corresponding teeth, as has just been stated, were removed after the operation. A probe inserted into these cavities shows that the bone contains rather shallow but well-marked depressions, which served for their lodgment. The free edges at the opening of these cavities have a ragged fringed appearance, indicating the ruptured attachments between the soft tissues and the teeth.

The remaining teeth now to be described are well shown in the illustration.

Tooth No. 1 is a second bicuspid, three-quarters of an inch long, with crown and fang complete. The masticating surface of the crown is about three-sixteenths of an inch in diameter, and possesses two cusps, of unequal length. The fang tapers to a point, and is half an inch in length. This tooth is of normal color, and is supplied with enamel, tartar, and cement, making it a perfectly developed tooth in every particular. It is firmly attached to the walls of the cyst by broad bands of connective tissue.

Tooth No. 2 is a wisdom tooth three-quarters of an inch long. Its crown possesses four cusps separated by a crucial depression. The fang is five-eighths of an inch long, transversely flattened, wedge shaped with no tendency to bifurcate. It also is attached to the wall of the cyst by connective tissue fibers.

Tooth No. 3 is a distinct cuspid, or canine. Its crown is conical, convex externally, concave internally, tapering to a blunted point. The neck is surrounded by a tissue resembling the normal gum. Its fang is firmly set into an irregular shaped mass, calcareous in color and appearance, but lacking the hardness and brittleness of calcareous structure. This mass, which is about five-eighths of an inch in diameter, is attached to the wall of the cyst by a short, thick, fleshy pedicle. Small pieces of this calcareous-like substance are scattered about the epidermal layer.

Hair follicles, sweat glands, nails, etc., were not found in connection with the inner layer.

From the description of its contents, this cyst is to be classified as dermoid, proliferative, dentigerous.

Interesting points about this cyst are the perfectly developed teeth which permit of classification, its large size, and the length of time it remained dormant in the system. WILLIAM C. KRAUSS.

## Figures and Tables

**Figure f1:**